# Target-Mediated Fluoroquinolone Resistance in *Neisseria gonorrhoeae*: Actions of Ciprofloxacin against Gyrase and Topoisomerase IV

**DOI:** 10.1021/acsinfecdis.4c00041

**Published:** 2024-03-04

**Authors:** Jessica A. Collins, Alexandria A. Oviatt, Pan F. Chan, Neil Osheroff

**Affiliations:** † Department of Biochemistry, Vanderbilt University School of Medicine, Nashville, Tennessee 37232, United States; ‡ Department of Medicine (Hematology/Oncology), Vanderbilt University School of Medicine, Nashville, Tennessee 37232, United States; § Infectious Diseases Research Unit, GlaxoSmithKline, Collegeville, Pennsylvania 19426, United States

**Keywords:** gyrase, topoisomerase IV, ciprofloxacin, fluoroquinolone, DNA cleavage, DNA supercoiling/decatenation

## Abstract

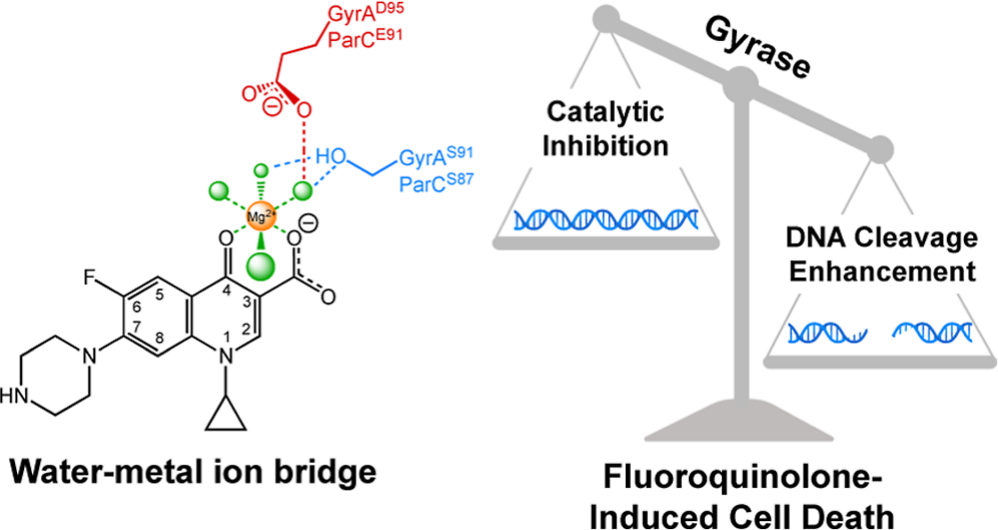

Fluoroquinolones make up a critically important class of antibacterials administered worldwide to treat human infections. However, their clinical utility has been curtailed by target-mediated resistance, which is caused by mutations in the fluoroquinolone targets, gyrase and topoisomerase IV. An important pathogen that has been affected by this resistance is *Neisseria gonorrhoeae*, the causative agent of gonorrhea. Over 82 million new cases of this sexually transmitted infection were reported globally in 2020. Despite the impact of fluoroquinolone resistance on gonorrhea treatment, little is known about the interactions of this drug class with its targets in this bacterium. Therefore, we investigated the effects of the fluoroquinolone ciprofloxacin on the catalytic and DNA cleavage activities of wild-type gyrase and topoisomerase IV and the corresponding enzymes that harbor mutations associated with cellular and clinical resistance to fluoroquinolones. Results indicate that ciprofloxacin interacts with both gyrase (its primary target) and topoisomerase IV (its secondary target) through a water–metal ion bridge that has been described in other species. Moreover, mutations in amino acid residues that anchor this bridge diminish the susceptibility of the enzymes for the drug, leading to fluoroquinolone resistance. Results further suggest that ciprofloxacin primarily induces its cytotoxic effects by enhancing gyrase-mediated DNA cleavage as opposed to inhibiting the DNA supercoiling activity of the enzyme. In conclusion, this work links the effects of ciprofloxacin on wild-type and resistant gyrase to results reported for cellular and clinical studies and provides a mechanistic explanation for the targeting and resistance of fluoroquinolones in *N. gonorrhoeae*.

Fluoroquinolones are one of the most important classes of antibacterial drugs in clinical use.^1−5^ High oral bioavailability, widespread tissue distribution, and broad-spectrum activity have led to the success of this class in treating a variety of Gram-positive and Gram-negative bacterial infections in humans.^1−3,5^ In 2019, more than 21 million prescriptions for fluoroquinolones were filled in the United States, and members of this antibacterial class are used heavily worldwide.^1,3,5^ The World Health Organization (WHO) lists fluoroquinolones as one of their five “highest priority” critically important antimicrobials for human medicine.^4^

Ciprofloxacin and other members of the fluoroquinolone class target the bacterial type II topoisomerases, gyrase and topoisomerase IV.^6−9^ Gyrase maintains the superhelical state of the bacterial genome and relieves torsional stress generated ahead of replication forks and transcription complexes.^10−16^ Topoisomerase IV can relax DNA supercoils but primarily unlinks (decatenates) replicated daughter chromosomes and removes knots from the genetic material.^10−17^ These type II enzymes capture, bend, and cleave a DNA segment and carry out their essential catalytic functions by passing a separate double helix through this transient double-stranded break.^6,12,14,15,18−21^ To initiate the DNA cleavage reaction, active site tyrosine residues launch a nucleophilic attack on the DNA backbone, generating a covalent linkage between the enzyme and the newly created 5′-terminal phosphate of the cleaved DNA.^6,12,14,15,19^ This covalent enzyme-cleaved DNA complex is known as the “cleavage complex”.^6,8,12,14,15,19,20^

Fluoroquinolones stabilize the cleavage complex by inserting into the cleaved scissile bonds on both strands of the DNA (one drug molecule per DNA strand), thereby inhibiting DNA ligation and increasing levels of DNA scission.^3,6−9,22^ When replication forks, transcription complexes, and other DNA tracking machinery approach drug-stabilized gyrase/topoisomerase IV-DNA complexes, the cut DNA can no longer be rejoined by the type II enzymes and is converted into persistent chromosomal breaks.^6,8,14,16,23,24^ If DNA recombination and repair processes cannot resolve these DNA breaks, the bacteria initiate cell death pathways.^8,9,25−27^ This mechanism of cytotoxicity is supported by the induction of the SOS DNA damage response in cells treated with fluoroquinolones.^9,26,28^

Because fluoroquinolones impair gyrase/topoisomerase IV-mediated ligation, they also inhibit enzyme catalysis and rob the cell of the essential functions of the type II enzymes.^8,9,13,14^ Decreased gyrase or topoisomerase IV activity can affect multiple nucleic acid processes, including DNA replication and daughter chromosome segregation, which slows cell growth and can lead to bacterial death.^6,8,25^ This second mechanism of cytotoxicity is supported by the fact that *Mycobacterium tuberculosis* hypomorphs that express reduced levels of DNA gyrase are hypersensitive to fluoroquinolones.^29^ At present, the relative contributions of DNA cleavage enhancement and catalytic inhibition to fluoroquinolone-induced cell death are poorly understood.

As determined by structural and biochemical studies, the primary conduit between fluoroquinolones and gyrase/topoisomerase IV is a water–metal ion bridge (Figure 1).^6,8,22,30−33^ The bridge starts with the C3/C4 keto acid of the fluoroquinolone, which chelates a noncatalytic divalent metal ion that is coordinated by four water molecules.^6,8,22,32,33^ Two of these water molecules hydrogen bond with a highly conserved serine (originally identified as Ser83 in *Escherichia coli* GyrA) and an acidic residue four amino acids upstream.^6,8,22,32,34,35^ Although this water–metal ion bridge appears to be a universal feature of fluoroquinolone–gyrase/topoisomerase IV interactions, utilization of the bridge varies from enzyme to enzyme and species to species.^6,8^ In some cases, both residues contribute strongly to drug interactions, while in others, either the serine or the acidic residue serves as the predominant anchor.^6,8,30,31,36−38^ Furthermore, the bridge can play important roles in either binding or positioning the fluoroquinolone.^6,8,30,31,36−38^ It is notable that the most important and prevalent mechanism of fluoroquinolone resistance is target-mediated and is caused by mutations in the two amino acids that anchor the water–metal ion bridge to the enzyme.^3,6−9,39^

**Figure 1:**
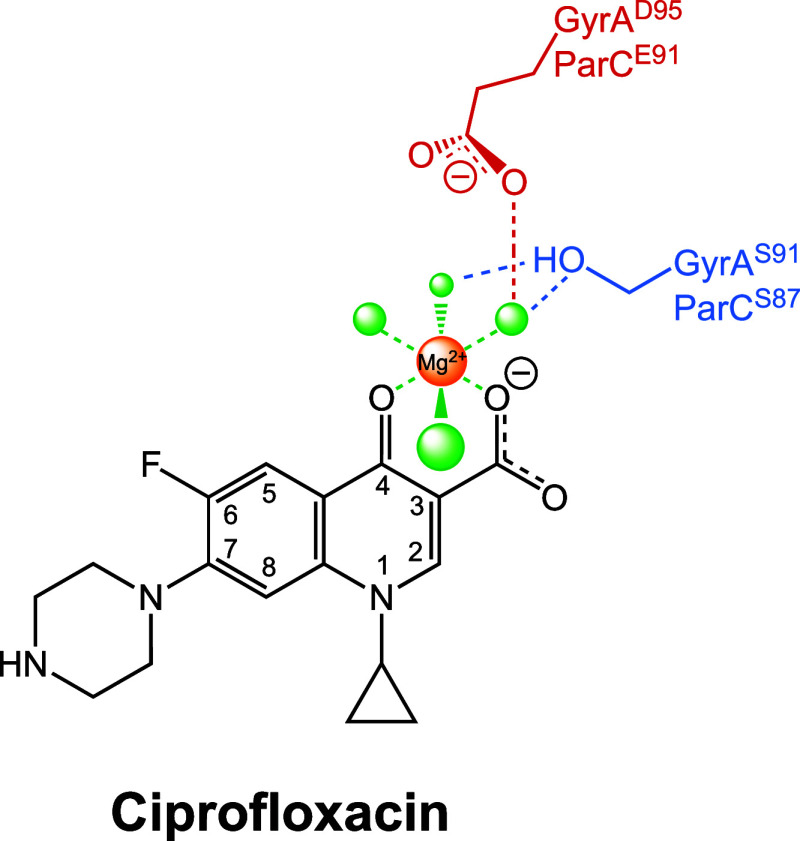
Schematic of the water–metal ion bridge that mediates interactions between fluoroquinolones and bacterial type II topoisomerases. Residue numbering reflects that of *N. gonorrhoeae* gyrase (GyrA) and topoisomerase IV (ParC). For simplicity, only interactions with the protein (and not DNA) are shown. A noncatalytic divalent metal ion (orange, Mg^2+^) forms an octahedral coordination sphere (green dashed lines) between four water molecules (green) and the C3/C4 keto acid of ciprofloxacin (black). Two of the water molecules form hydrogen bonds (blue dashed lines) with the serine side chain hydroxyl group (blue), and one water molecule forms hydrogen bonds (red dashed lines) with the aspartic acid (GyrA) or glutamic acid (ParC) side chain carboxyl group (red).

Since fluoroquinolones were first introduced into the clinic, high levels of target-mediated resistance have curtailed the clinical use of this class to treat some bacterial infections.^3,6,8,9^ An important example is *Neisseria gonorrhoeae*, a Gram-negative bacterium that is the etiological agent of gonorrhea.^40^ This sexually transmitted disease infects the mucosal epithelium of the genitals, rectum, and throat and causes more than 82 million new cases globally each year.^41,42^ If left untreated, gonorrhea can cause severe complications including pelvic inflammatory disease and infertility, and when disseminated, infections can result in death.^41,43^

Ciprofloxacin was introduced as frontline treatment for gonorrheal infections in the early 1990s.^44,45^ However, the drug was removed from treatment guidelines for this indication by the Centers for Disease Control and Prevention (CDC) in 2006 due to high levels of resistance.^46^ In 2021, nearly 33% of clinical *N. gonorrhoeae* isolates in the United States were resistant to ciprofloxacin compared with 13.3% in 2011 and 0.7% in 2001.^47^ As a result of resistance to fluoroquinolones and other antibacterials, gonorrhea is listed as one of five “urgent threats” (the highest threat level) for resistance by the CDC,^48^ and the WHO has warned that drug-resistant gonorrhea has the potential to become the third incurable sexually transmitted disease following HIV/AIDS and herpes.^49^

Despite the prevalence and clinical impact of fluoroquinolone-resistant gonorrhea, little is known about the interactions of this drug class with its type II topoisomerase targets from *N. gonorrhoeae*. Therefore, we characterized the effects of ciprofloxacin on the catalytic and DNA cleavage activities of gyrase and topoisomerase IV from this species. Interactions with wild-type (WT) enzymes and with enzymes harboring mutations found in fluoroquinolone-resistant isolates were determined. Results suggest that the enhancement of gyrase-mediated DNA cleavage is the primary mechanism by which ciprofloxacin induces its cytotoxic effects. Furthermore, the in vitro effects of mutations in residues that anchor the water–metal ion bridge in gyrase and topoisomerase IV may explain the patterns of enzyme targeting and clinical resistance to fluoroquinolones in this sexually transmitted disease.

## Results and Discussion

### Gyrase-Mediated Fluoroquinolone Resistance

Shortly after fluoroquinolones were approved to treat gonorrhea, cases of drug resistance were reported.^45,50−54^ In laboratory experiments utilizing cultured strains of *N. gonorrhoeae* serially diluted and plated with increasing concentrations of ciprofloxacin, the first spontaneous mutations that exhibited decreased susceptibility to fluoroquinolones were seen in gyrase.^55^ This result indicated that gyrase is the primary cellular target for ciprofloxacin and (presumably) other members of this drug class in gonorrhea.^55,56^ Similar results have been reported for resistant isolates from clinical samples.^57−61^

Overwhelmingly, mutations in the GyrA subunit of gyrase are observed at Ser91 and Asp95 in *N. gonorrhoeae*.^62^ In laboratory-based genetic studies, the initial mutation event occurs at Ser91, followed by the acquisition of a second genetic alteration at Asp95 to yield a double mutant.^55^ Individual substitution at Asp95 is rarely found.^55,62,63^ Furthermore, higher MIC (minimum inhibitory concentration) values for ciprofloxacin in laboratory strains and clinical isolates are associated with gyrase that contains mutations in both residues.^55,58,59,61^ These findings are reflected in the prevalence of the mutations in patient samples, in which 77.0% of resistant isolates harbor mutations at both Ser91 and Asp95, as opposed to 19.0% at Ser91 and only 2.5% at Asp95 individually.^62^ The serine and aspartic acid associated with fluoroquinolone resistance are the residues predicted to anchor the fluoroquinolone–gyrase–water–metal ion bridge shown in Figure 1.^6,8,22,32,33,62^ These findings suggest that *N. gonorrhoeae* gyrase, like other species, relies on the water–metal ion bridge to facilitate interactions with fluoroquinolones.

Despite the importance of gyrase as the primary target of fluoroquinolone treatment in gonorrhea and the role of Ser91 and Asp95 in drug resistance, very little has been reported regarding the interactions of fluoroquinolones with this type II enzyme from *N. gonorrhoeae*. Moreover, the contributions of the individual amino acid residues to bridge function have never been reported, as only the double mutant has been assessed.^28^ Therefore, we investigated the effects of ciprofloxacin on the catalytic and DNA cleavage activities of WT gyrase, as well as the individual (GyrA^S91F^ and GyrA^D95G^) and double (GyrA^S91F/D95G^) mutants associated with fluoroquinolone resistance in cellular and clinical studies.

**Figure 2:**
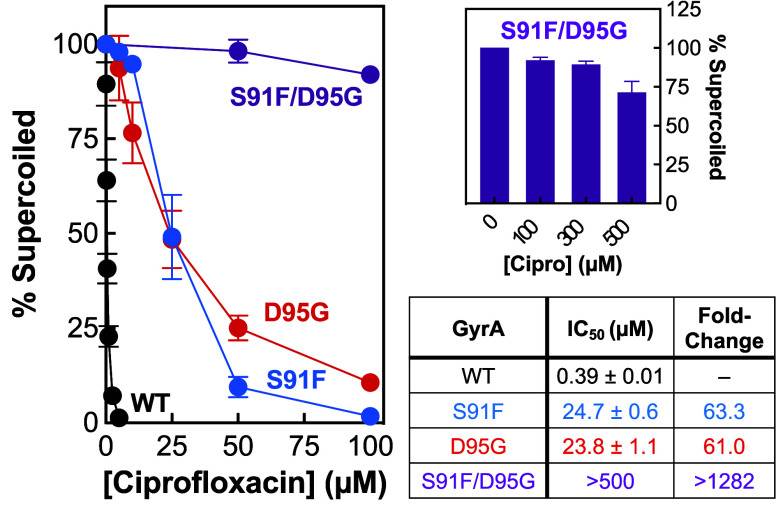
Effects of ciprofloxacin on DNA supercoiling catalyzed by WT and mutant *N. gonorrhoeae* gyrase. The abilities of WT (black), GyrA^S91F^ (S91F, blue), GyrA^D95G^ (D95G, red), and GyrA^S91F/D95G^ (S91F/D95G, purple) gyrase to supercoil relaxed plasmids in the presence of ciprofloxacin are shown in the left panel. The right-hand panel displays the ability of GyrA^S91F/D95G^ gyrase to supercoil relaxed DNA at high ciprofloxacin concentrations (up to 500 μM). Error bars represent the standard deviation of at least 3 independent experiments. The table indicates the corresponding IC_50_ values (the drug concentration at which the enzyme activity is inhibited by 50%), including the standard error of the mean and the fold-change in IC_50_ from WT.

Initial studies examined the effects of ciprofloxacin on catalytic activity by monitoring DNA supercoiling catalyzed by WT, GyrA^S91F^, GyrA^D95G^, and GyrA^S91F/D95G^*N. gonorrhoeae* gyrase (Figures 2 and S1). The fluoroquinolone was a potent inhibitor of the WT enzyme with an IC_50_ value of 0.39 μM. Despite the fact that mutations at Ser91 are much more prevalent than those at Asp95 in clinical isolates,^62^ ciprofloxacin showed equivalent reductions in potency (∼60-fold) against the GyrA^S91F^ and GyrA^D95G^ mutant enzymes (IC_50_ values of 24.7 and 23.8 μM, respectively). Consistent with cellular results,^55,59,62^ the GyrA^S91F/D95G^ double mutant showed a considerably reduced susceptibility to ciprofloxacin (Figure 2, right). Even at 500 μM drug, less than 30% inhibition of DNA supercoiling was observed.

**Figure 3:**
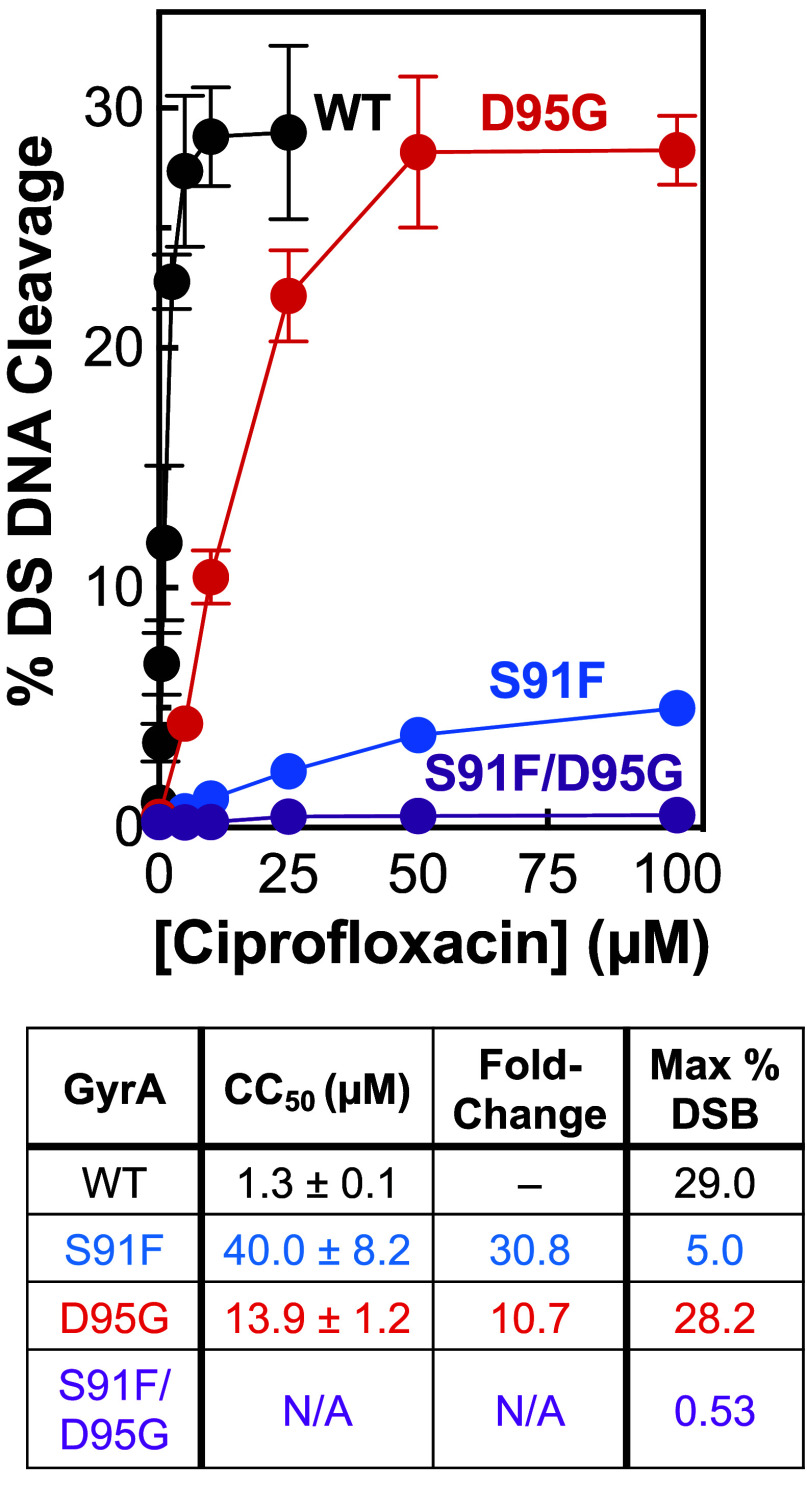
Effects of ciprofloxacin on DNA cleavage mediated by WT and mutant *N. gonorrhoeae* gyrase. The ability of ciprofloxacin to induce double-stranded (DS) DNA cleavage mediated by WT (black), GyrA^S91F^ (S91F, blue), GyrA^D95G^ (D95G, red), and GyrA^S91F/D95G^ (S91F/D95G, purple) gyrase is shown in the top panel. Error bars represent the standard deviation of at least 3 independent experiments. The table at the bottom lists the CC_50_ value (the drug concentration at which 50% maximal DS DNA cleavage is reached) for each enzyme, including the standard error of the mean, the fold-change in CC_50_ from WT, and the max % DSB value (maximal percentage of DS DNA breaks) induced at 100 μM ciprofloxacin. Values marked as N/A (Not Analyzed) were excluded from additional analyses due to low signal.

Considering that GyrA^S91F^ and GyrA^D95G^ have similar sensitivities to ciprofloxacin in DNA supercoiling assays, it is unclear why individual mutations at Asp95 are rarely observed in resistant isolates, while those at Ser91 occur at high frequencies.^62^ Consequently, we compared the effects of ciprofloxacin on DNA cleavage mediated by gyrase harboring the GyrA^S91F^ and GyrA^D95G^ single mutations to those of WT *N. gonorrhoeae* gyrase (Figures 3 and S2). In contrast to DNA supercoiling, there was a striking difference between the two mutants in the DNA cleavage assays. Ciprofloxacin increased double-stranded DNA scission mediated by GyrA^D95G^ to a level comparable to that of WT gyrase (28.2 vs 29.0%, respectively), albeit with an ∼10-fold reduction in potency (CC_50_ values of 13.9 vs 1.3 μM, respectively). However, even at 100 μM, ciprofloxacin induced no more than 5.0% DNA cleavage with GyrA^S91F^ gyrase with an even greater reduction in potency (CC_50_ = 40.0 μM). These results strongly suggest that ciprofloxacin resistance in *N. gonorrhoeae* cells that carry the GyrA^S91F^ mutation tracks with gyrase-mediated DNA cleavage rather than gyrase-catalyzed DNA supercoiling. They further imply that under normal growth conditions, fluoroquinolone-induced cytotoxicity associated with this first-step mutation is caused by the introduction of breaks in the bacterial chromosome as opposed to the loss of gyrase function.

The reduced susceptibility of GyrA^S91F/D95G^ to ciprofloxacin was far more dramatic than that seen with either of the enzymes carrying the single mutations (Figures 3 and S2). Even at concentrations as high as 500 μM (Figure S2), the fluoroquinolone induced only 0.53% double-stranded DNA cleavage. This finding is consistent with clinical studies in which the double mutant is considerably more prevalent than the single Ser91 mutant in resistant strains.^62^ Although the enhanced resistance associated with the GyrA^S91F/D95G^ mutation may be attributed to further loss of DNA scission induced by ciprofloxacin, it is tempting to speculate that the inability of the drug to inhibit DNA supercoiling by the double mutant (see Figure 2) may contribute to its reduced susceptibility to fluoroquinolones in cells that harbor this double mutant gyrase.

### Use of the Water–Metal Ion Bridge to Promote Fluoroquinolone Interactions in *N. gonorrhoeae* Gyrase

Previous studies provide strong evidence that the water–metal ion bridge is the primary conduit between fluoroquinolones and bacterial type II topoisomerases (see Figure 1) but that the amino acids that anchor the bridge are utilized differently across species and enzymes.^6,8,30,31,36−38^ The finding that mutations at Ser91 and Asp95 comparably reduce the IC_50_ values for inhibition of DNA supercoiling (see Figure 2) and also lessen ciprofloxacin potency in DNA cleavage assays (see Figure 3) suggests that these two bridge-anchoring residues contribute similarly to the affinity of *N. gonorrhoeae* gyrase for ciprofloxacin. However, the dramatic decrease in drug-induced DNA cleavage by GyrA^S91F^ implies that Ser91 (in contrast to Asp95) also plays a role in correctly positioning ciprofloxacin in the active site of the enzyme such that it stabilizes the cleavage complex.

**Figure 4:**
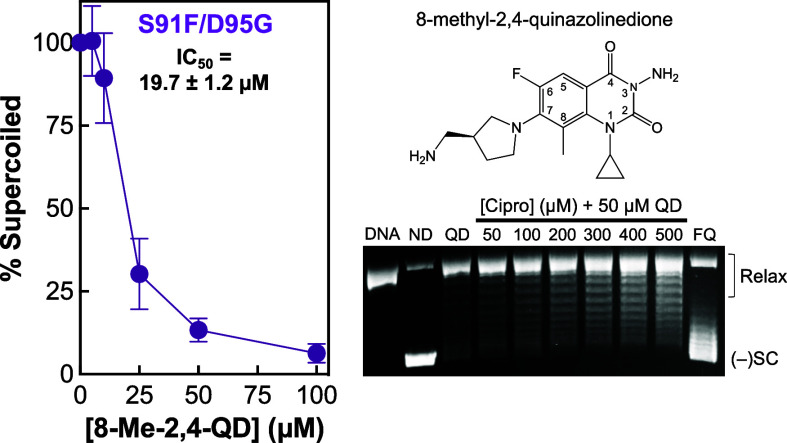
Effects of 8-methyl-2,4-quinazolinedione on the DNA supercoiling activities of GyrA^S91F/D95G^*N. gonorrhoeae* gyrase. The ability of 8-methyl-2,4-quinazolinedione (8-Me-2,4-QD, structure at the right) to inhibit DNA supercoiling catalyzed by GyrA^S91F/D95G^ gyrase (purple) is shown in the left panel (IC_50_ = 19.7 ± 1.2 μM). Error bars represent the standard deviation of at least 3 independent experiments. The ability of 0–500 μM ciprofloxacin to compete with 50 μM 8-methyl-2,4-quinazolinedione (QD) for GyrA^S91F/D95G^ gyrase during DNA supercoiling is shown in the gel in the right panel. Reaction mixtures contained DNA in the absence of the enzyme (DNA) or GyrA^S91F/D95G^ gyrase in the absence of the compound (ND, no drug), in the presence of either 50 μM 8-methyl-2,4-quinazolinedione (QD) or 500 μM ciprofloxacin (FQ) alone or in the presence of 50 μM QD and increasing concentrations of ciprofloxacin (cipro, 50–500 μM). Both drugs were added to reaction mixtures simultaneously. The positions of the relaxed (Relax) and negatively supercoiled [(−)SC] plasmids are indicated. The gel is representative of 3 independent experiments.

The reduced effects of ciprofloxacin on GyrA^S91F/D95G^ compared to those on the single mutant enzymes (GyrA^S91F^ and GyrA^D95G^) strongly suggest that simultaneous substitution of both bridge-anchoring residues severely diminishes the affinity of the enzyme for the fluoroquinolone. To determine whether this was the case, competition studies that monitored fluoroquinolone interactions at the site of the water−metal ion bridge were performed. To this end, the ability of ciprofloxacin to compete with 8-methyl-2,4-quinazolinedione was assessed (Figure 4). Quinazolinediones are similar in structure to fluoroquinolones but lack the C3/C4 keto acid that is required to chelate the divalent metal ion used in the bridge.^64,65^ Although they share a binding site with fluoroquinolones,^20,66^ 8-methyl-2,4-quinazolinedione and related compounds interact with bacterial type II topoisomerases through their C7 3′-(aminomethyl)pyrrolidinyl moiety, independent of the water–metal ion bridge.^20,64,65,67,68^ Consequently, quinazolinediones generally show high activity against fluoroquinolone-resistant type II topoisomerases.^6,8,69^

As seen in Figure 4, 8-methyl-2,4-quinazolinedione inhibits DNA supercoiling catalyzed by GyrA^S91F/D95G^ with an IC_50_ of 19.7 μM. Because ciprofloxacin has virtually no effect on supercoiling catalyzed by the GyrA^S91F/D95G^ double mutant enzyme (see Figure 2), if it effectively competes for enzyme binding with 8-methyl-2,4-quinazolinedione, the fluoroquinolone should overcome the ability of the quinazolinedione to inhibit this enzyme-catalyzed reaction. However, even at concentrations as high as 500 μM, ciprofloxacin showed little ability to reverse the inhibition of DNA supercoiling at a near-saturating concentration of quinazolinedione (see the gel in Figure 4). This indicates that the affinity of ciprofloxacin for GyrA^S91F/D95G^ gyrase is greatly reduced compared to that of the WT enzyme.

**Figure 5:**
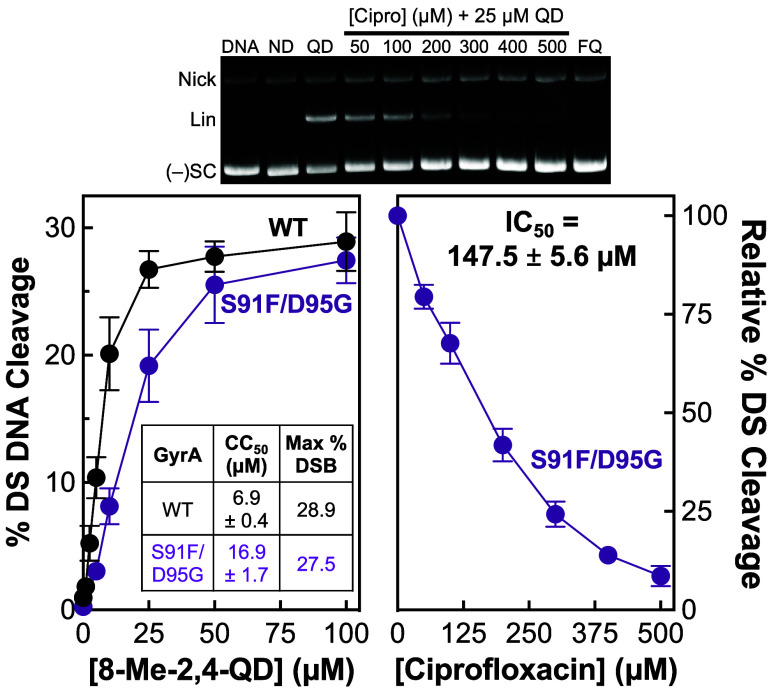
Effects of 8-methyl-2,4-quinazolinedione on the DNA cleavage activities of the WT and GyrA^S91F/D95G^ gyrase. The ability of 8-methyl-2,4-quinazolinedione (8-me-2,4-QD) to induce double-stranded (DS) DNA cleavage mediated by WT (black) and GyrA^S91F/D95G^ gyrase (S91F/D95G, purple) is shown in the left panel. The inset table shows the corresponding CC_50_, including the standard error of the mean and max % DSB values. The ability of 0–500 μM ciprofloxacin (Cipro) to compete with 25 μM 8-methyl-2,4-quinazolinedione (QD) for GyrA^S91F/D95G^ gyrase-mediated DNA cleavage is shown in the right panel, including the IC_50_ value. Both drugs were added to reaction mixtures simultaneously. The relative contribution of the quinazolinedione to the total level of DNA cleavage was calculated as follows: (DS DNA cleavage in the presence of quinazolinedione and fluoroquinolone—DS DNA cleavage in the absence of either compound)/(DS DNA cleavage in the presence of 25 μM quinazolinedione only). Error bars represent the standard deviation of at least 3 independent experiments. A gel image displaying the competition data quantified in the right panel is shown at the top. Reaction mixtures contained DNA in the absence of the enzyme (DNA) or GyrA^S91F/D95G^ gyrase in the absence of the compound (ND, no drug), in the presence of either 25 μM 8-methyl-2,4-quinazolinedione (QD) or 500 μM ciprofloxacin (FQ) alone, or in the presence of 25 μM 8-methyl-2,4-quinazolinedione (QD) and increasing concentrations of ciprofloxacin (Cipro, 50–500 μM). The positions of nicked (Nick), linear (Lin), and negatively supercoiled [(−)SC] plasmids are indicated. The gel is representative of 3 independent experiments.

To further study the effects of the double mutation on fluoroquinolone–gyrase interactions, we examined the competition between ciprofloxacin and 8-methyl-2,4-quinazolinedione in DNA cleavage assays. As seen in Figure 5 (left panel), the quinazolinedione maintained (compared to that of the WT enzyme) a high affinity and a high maximal level of DNA cleavage with GyrA^S91F/D95G^. In contrast, ciprofloxacin did not enhance DNA scission at 500 μM with GyrA^S91F/D95G^ (Figure 5, right, FQ lane, and Figure S2). Thus, competition was monitored by the loss of quinazolinedione-induced double-stranded DNA breaks. Although ciprofloxacin was able to compete with 8-methyl-2,4-quinazolinedione, its competition IC_50_ value was ∼10-fold higher than the CC_50_ value of the quinazolinedione against the double mutant enzyme and more than 100 times higher than its CC_50_ value against WT gyrase (see Figure 3). Taken together, these studies bolster the conclusion that the loss of the water–metal ion bridge substantially impedes the interactions of ciprofloxacin with GyrA^S91F/D95G^. These results provide an overarching mechanism for the high levels of drug resistance observed in strains that carry gyrase mutations in both Ser91 and Asp95.

### Topoisomerase IV-Mediated Fluoroquinolone Resistance

Although gyrase is the primary cellular target for fluoroquinolones in *N. gonorrhoeae*, laboratory strains and clinical isolates that carry additional mutations in topoisomerase IV show even higher levels of resistance.^55,57−61,70−73^ These findings indicate that topoisomerase IV is a secondary target for this drug class in *N. gonorrhoeae*.^45,56^ Therefore, we evaluated the effects of ciprofloxacin on WT topoisomerase IV and enzymes that contained the single ParC^S87N^ or ParC^E91G^ mutation or the double ParC^S87N/E91G^ mutation. These substitutions are associated with fluoroquinolone resistance in cellular and clinical studies and occur at the residues predicted to anchor the water–metal ion bridge in *N. gonorrhoeae* topoisomerase IV.^6,8,30,31,36−38,63^

**Figure 6:**
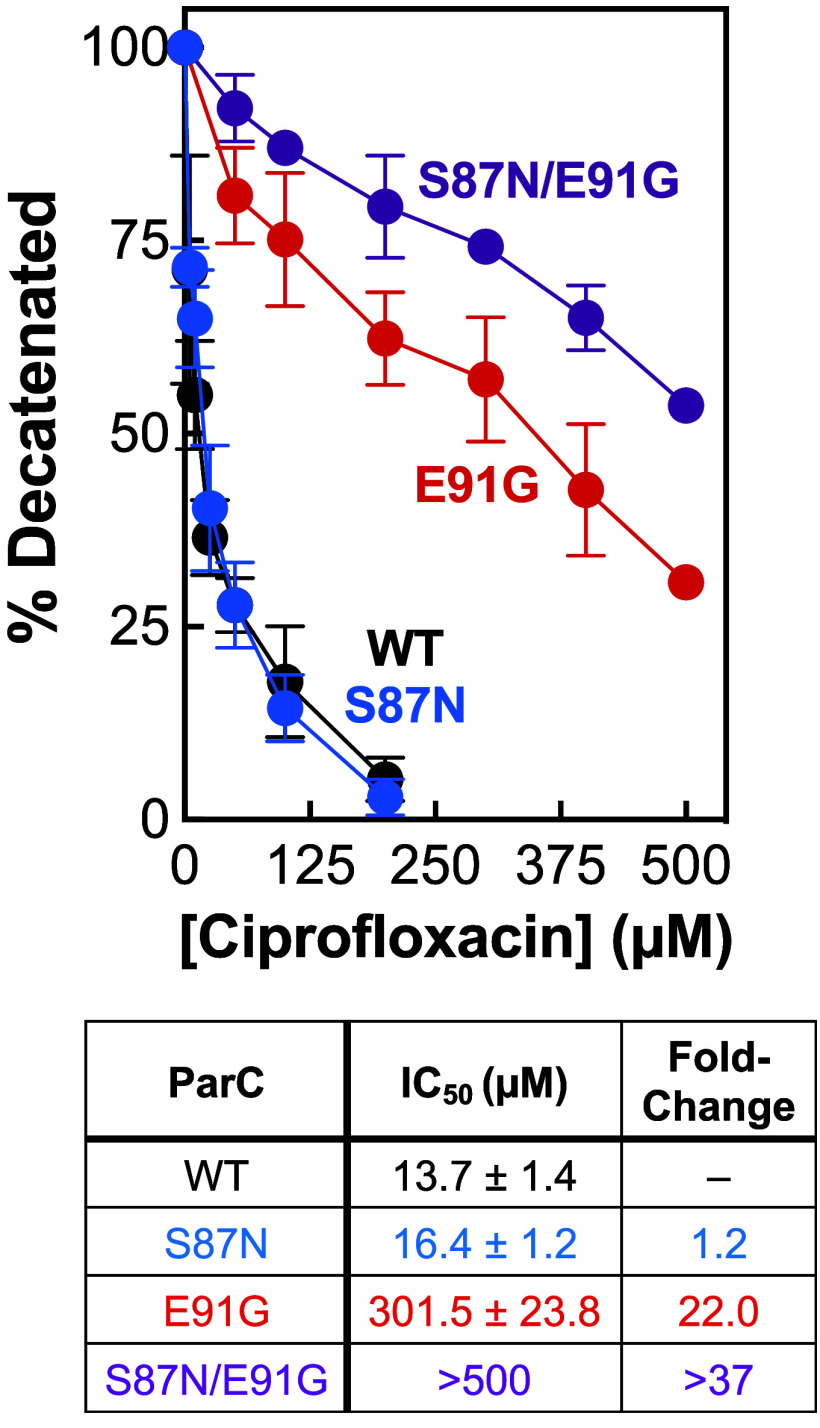
Effects of ciprofloxacin on the DNA decatenation activities of WT and mutant *N. gonorrhoeae* topoisomerase IV. The ability of ciprofloxacin to inhibit decatenation catalyzed by WT (black), ParC^S87N^ (S87N, blue), ParC^E91G^ (E91G, red), and ParC^S87N/E91G^ (S87N/E91G, purple) topoisomerase IV is shown in the top panel. Error bars represent the standard deviations of at least 3 independent experiments. Corresponding IC_50_ values, including the standard error of the mean and the fold-change in IC_50_ from WT, are indicated in the table at the bottom.

Initial experiments examined the effects of ciprofloxacin on topoisomerase IV-catalyzed decatenation (Figures 6 and S3). The fluoroquinolone was a less potent catalytic inhibitor against WT topoisomerase IV (IC_50_ = 13.7 μM) than it was for gyrase (IC_50_ = 0.39 μM, see Figure 2).

Furthermore, the mutations in the predicted bridge-anchoring residues had a smaller effect on the susceptibility of topoisomerase IV to ciprofloxacin than that observed with gyrase. As compared to WT topoisomerase IV, the ParC^S87N^ mutation had virtually no effect on the sensitivity of the enzyme to ciprofloxacin (IC_50_ = 16.4 μM). Moreover, the difference in the relative sensitivity between the WT enzyme and ParC^E91G^ or ParC^S87N/E91G^ was also smaller than that observed for the mutations at analogous residues in gyrase (see Figure 2).

**Figure 7:**
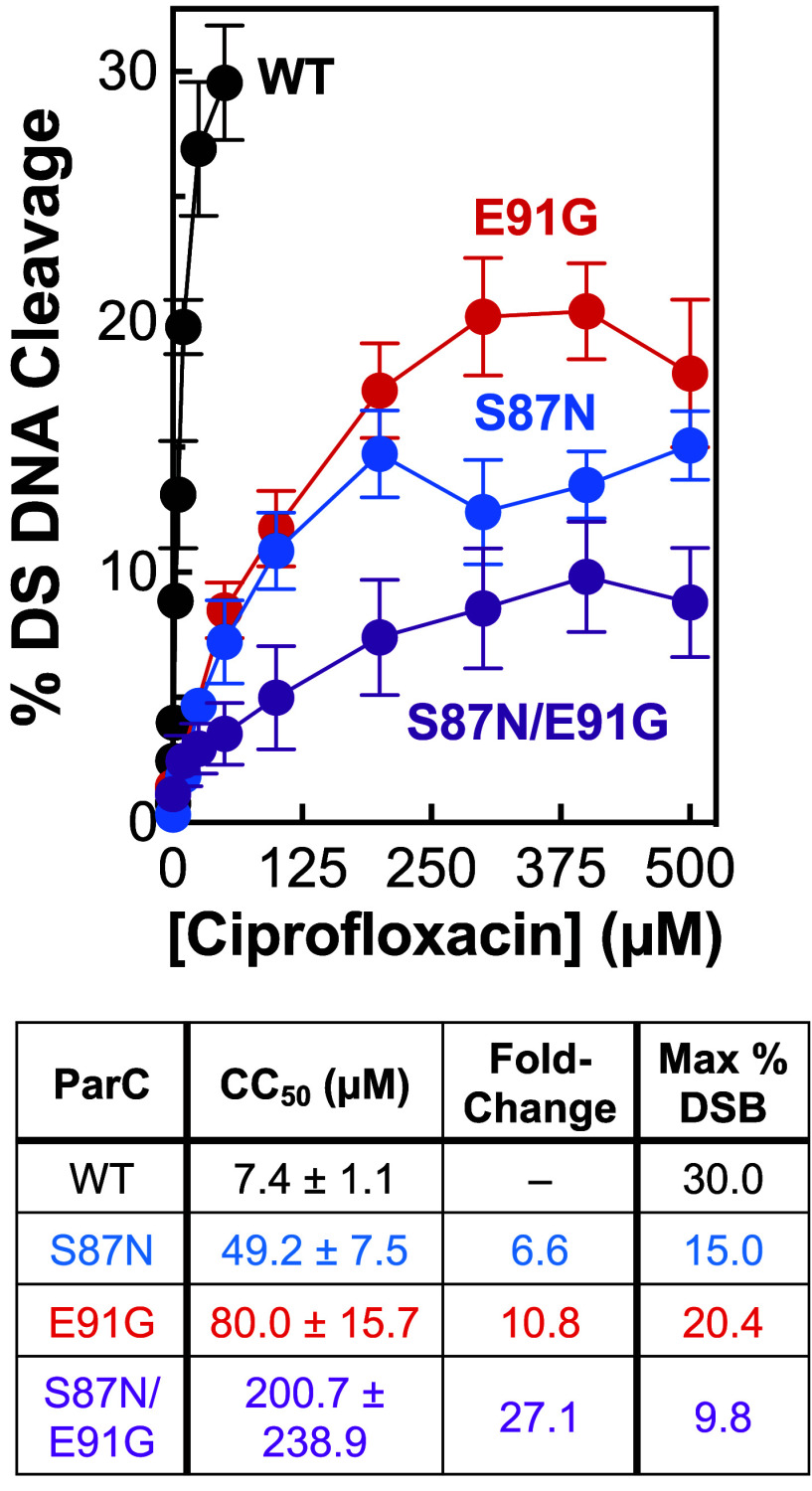
Effects of ciprofloxacin on the DNA cleavage activities of WT and mutant *N. gonorrhoeae* topoisomerase IV. The ability of ciprofloxacin to induce double-stranded (DS) DNA cleavage mediated by WT (black), ParC^S87N^ (S87N, blue), ParC^E91G^ (E91G, red), and ParC^S87N/E91G^ (S87N/E91G, purple) topoisomerase IV is displayed in the top panel. Error bars represent the standard deviations of at least 3 independent experiments. The corresponding CC_50_ values, including the standard error of the mean, fold-change in CC_50_ from WT, and max % DSB values, are indicated in the table at the bottom.

Further experiments investigated the effects of ciprofloxacin on DNA cleavage mediated by WT and mutant *N. gonorrhoeae* topoisomerase IV (Figures 7 and S4). Once again, the fluoroquinolone appeared to be less potent against WT topoisomerase IV (CC_50_ = 7.4 μM) than against gyrase (CC_50_ = 1.3 μM, see Figure 3). Levels of DNA scission mediated by WT topoisomerase IV above 50 μM ciprofloxacin are not included in Figure 7 as multiple cleavage events per plasmid were observed at higher drug concentrations (Figure S4). Thus, the actual CC_50_ value is likely to be higher than the calculated value. Even though the ParC^S87N^ mutation had no effect on the susceptibility of topoisomerase IV to ciprofloxacin in decatenation assays, it affected both the potency (CC_50_ = 49.2 μM) and efficacy (max % DSB = 15.0) of the drug in cleavage assays. Additionally, topoisomerase IV containing the ParC^S87N/E91G^ double mutation exhibited considerably higher levels of fluoroquinolone-induced cleavage activity (max % DSB = 9.8) than was seen with gyrase (max % DSB at 500 μM ciprofloxacin = 0.53).

Taken together, the catalytic and DNA cleavage activities of *N. gonorrhoeae* topoisomerase IV suggest that, similar to gyrase, the mutation at ParC Ser87 affects the positioning of the fluoroquinolone in the active site, reducing its ability to stabilize cleavage complexes. However, unlike results with gyrase, individual mutations at bridge-anchoring residues (ParC^S87N^ and ParC^E91G^) had a similar effect on ciprofloxacin sensitivity in DNA cleavage assays. This implies a greater role for the acidic residue in anchoring topoisomerase IV-fluoroquinolone interactions than was observed with gyrase.

Unfortunately, the relatively high activity of ciprofloxacin against fluoroquinolone-resistant topoisomerase IV precluded the use of quinazolinedione competition studies to further analyze the role of the water–metal ion bridge in mediating drug–enzyme interactions. However, as compared with gyrase, the catalytic and DNA cleavage data for WT and mutant topoisomerase IV that harbor residues associated with fluoroquinolone resistance indicate that ciprofloxacin is less potent against topoisomerase IV and that mutations at bridge-anchored residues have a lesser effect on the sensitivity of the enzyme to ciprofloxacin. Taken together, these findings may explain (at least in part) why topoisomerase IV is the secondary target of fluoroquinolones in *N. gonorrhoeae*.

Finally, the number of clinical studies that report single mutations at either ParC^S87N^ or ParC^E91G^ in topoisomerase IV is limited, and conclusions are complicated by the fact that these residues are only observed in the background of fluoroquinolone resistance mutations in gyrase.^57,59,61,71,73^ Thus, further studies will be necessary to draw conclusions regarding the relative importance of mutations at the two bridge-anchoring residues in *N. gonorrhoeae* topoisomerase IV to clinical resistance.

In summary, fluoroquinolone-resistant *N. gonorrhoeae* is an immediate threat to global health. Unfortunately, little is known about the interactions of fluoroquinolones with gyrase and topoisomerase IV from this species. The work presented above links the effects of ciprofloxacin on gyrase to cellular and clinical studies and provides a mechanistic underpinning for the targeting and resistance of fluoroquinolones in *N. gonorrhoeae*.

## Materials and Methods

### DNA, Materials, and Enzymes

Negatively supercoiled pBR322 DNA was prepared from *E. coli* using a Plasmid Mega Kit (Qiagen) as described by the manufacturer. Relaxed pBR322 plasmid was generated by treating negatively supercoiled pBR322 with calf thymus topoisomerase I (Invitrogen) in 50 mM Tris–HCl (pH 7.5), 50 mM KCl, 10 mM MgCl_2_, 0.5 mM DTT, 0.1 mM EDTA, and 30 μg/mL bovine serum albumin (BSA) for 45 min at 37 °C, followed by heat inactivation of topoisomerase I at 75 °C for 10 min.^74^ Kinetoplast DNA (kDNA) was isolated from *Crithidia fasciculata,* as described by Englund.^75^

Ciprofloxacin (Sigma) was stored at −20 °C as a 40 mM stock solution in 0.1 M NaOH and diluted 5-fold with 10 mM Tris–HCl (pH 7.9) immediately prior to use. 3-Amino-7-[(3*S*)-3-(aminomethyl)-1-pyrrolidinyl]-1-cyclopropyl-6-fluoro-8-methyl-2,4(1*H*,3*H*)-quinazolinedione was synthesized using established methods, as reported previously, and was the gift of Dr. Robert Kerns.^76^ For the sake of simplicity, this compound will be termed 8-methyl-2,4-quinazolinedione. This compound was stored at −20 °C as 20 mM stock solutions in 100% DMSO. All other chemicals were of analytical reagent grade.

All proteins were His-tagged. *N. gonorrhoeae* WT gyrase (GyrA, GyrB) and topoisomerase IV (ParC, ParE) subunits as well as mutant GyrA^S91F^ and GyrA^S91F/D95G^ gyrase were prepared by GenScript, as described previously.^28,32,77^*N. gonorrhoeae* mutant GyrA^D95G^ gyrase and mutant ParC^S87N^, ParC^E91G^, and ParC^S87N/E91G^ topoisomerase IV were generated using a QuickChange II XL site-directed mutagenesis kit (Agilent Technologies) with custom primers for the desired mutations. Mutant *N. gonorrhoeae* GyrA and ParC subunits were expressed and purified as described by Ashley et al.^78^ with the following modifications to optimize protein expression and lysis: (1) GyrA^D95G^ was expressed for 2.5 h and ParC^S87N^, ParC^E91G^, and ParC^S87N/E91G^ were expressed for 3 h before harvesting and (2) cells were lysed by sonication using a digital sonifier (Branson). The identities of all constructs were confirmed by DNA sequencing, and all enzymes were stored at −80 °C. In all assays, *N. gonorrhoeae* gyrase or topoisomerase IV was used as a 1:1 GyrA/GyrB or ParC/ParE mixture, respectively, and the stated enzyme concentration reflects that of the holoenzyme (A_2_B_2_).

### Gyrase-Catalyzed DNA Supercoiling

DNA supercoiling assays were based on previously published protocols by Aldred et al.^37^ Assays contained 15 nM WT or 25 nM mutant (GyrA^S91F^, GyrA^D95G^, or GyrA^S91F/D95G^) *N. gonorrhoeae* gyrase, 5 nM relaxed pBR322, and 1.5 mM ATP in a total volume of 20 μL of 50 mM Tris–HCl (pH 7.5), 175 mM KGlu, 5 mM MgCl_2_, and 50 μg/mL BSA. Assay mixtures were incubated at 37 °C for 20 min with WT and GyrA^D95G^, 25 min with GyrA^S91F/D95G^, or 30 min with GyrA^S91F^*N. gonorrhoeae* gyrase, which represents the minimum time required to completely supercoil the DNA in the absence of a drug. Reactions were stopped by the addition of 3 μL of a mixture of 0.77% SDS and 77.5 mM Na_2_EDTA. Samples were mixed with 2 μL of loading dye [60% sucrose, 10 mM Tris–HCl (pH 7.9), 0.5% bromophenol blue, and 0.5% xylene cyanol FF] and incubated at 45 °C for 2 min before being subjected to electrophoresis on 1% agarose gels in 100 mM Tris-borate (pH 8.3) and 2 mM EDTA. Gels were stained with 1 μg/mL ethidium bromide for 20 min and then destained with distilled water for 10 min. DNA bands were visualized with medium-range ultraviolet light and quantified using an Alpha Innotech digital imaging system (Protein Simple). IC_50_ values (the concentration of drug required to inhibit enzyme activity by 50%) were calculated on GraphPad Prism Version 10.0.3 using a nonlinear regression analysis with 95% confidence intervals.

For assays that monitored competition between ciprofloxacin and 8-methyl-2,4-quinazolinedione, fluoroquinolone (0–500 μM) and quinazolinedione (50 μM) were added simultaneously to reaction mixtures.

### Topoisomerase IV-Catalyzed DNA Decatenation

DNA decatenation assays were based on previously published protocols by Anderson et al.^79^ and Aldred et al.^30^ Assays contained 10 nM WT, 20 nM ParC^S87N^, 35 nM ParC^E91G^, or 35 nM ParC^S87N/E91G^*N. gonorrhoeae* topoisomerase IV, 5 nM kDNA, and 1 mM ATP in 20 μL of 40 mM HEPES-KOH (pH 7.6), 25 mM NaCl, 100 mM KGlu, and 10 mM Mg(OAc)_2_. Assay mixtures were incubated at 37 °C for 10 min with WT, 15 min with ParC^S87N^, and 30 min with ParC^E91G^ and ParC^S87N/E91G^*N. gonorrhoeae* topoisomerase IV, which represents the minimum time required to completely decatenate the kDNA in the absence of a drug. Reactions were stopped, subjected to electrophoresis, and visualized as described for gyrase-catalyzed DNA supercoiling. IC_50_ values were calculated on GraphPad Prism Version 10.0.3 using a nonlinear regression analysis with 95% confidence intervals.

### DNA Cleavage

DNA cleavage reactions were performed according to the procedure of Aldred et al.^30^ Reactions were performed in the absence or presence of increasing concentrations of ciprofloxacin. Unless stated otherwise, assay mixtures contained 10 nM pBR322 and 100 nM WT, 100 nM GyrA^S91F^, 100 nM GyrA^D95G^, or 100 nM GyrA^S91F/D95G^*N. gonorrhoeae* gyrase or 100 nM WT, 200 nM ParC^S87N^, 150 nM ParC^E91G^, or 150 nM ParC^S87N/E91G^*N. gonorrhoeae* topoisomerase IV in a total volume of 20 μL of 40 mM Tris–HCl (pH 7.9), 50 mM NaCl, 2.5% (w/v) glycerol, and 10 mM MgCl_2_. The concentrations of mutant enzymes employed were normalized to provide the same level of cleavage as the WT enzyme in the presence of 8-methyl-2,4-quinazolinedione.

Reactions were incubated at 37 °C for 30 min with WT and mutant (GyrA^S91F^, GyrA^D95G^, and GyrA^S91F/D95G^) *N. gonorrhoeae* gyrase, 20 min with mutant (ParC^S87N^, ParC^E91G^, and ParC^S87N/E91G^) *N. gonorrhoeae* topoisomerase IV, and 10 min with WT *N. gonorrhoeae* topoisomerase IV. Enzyme-DNA cleavage complexes were trapped by adding 2 μL of 4% SDS, followed by 2 μL of 250 mM EDTA (pH 8.0). Proteinase K was added (2 μL of a 0.8 mg/mL solution), and the reaction mixtures were incubated at 45 °C for 30 min to digest the enzyme. Samples were mixed with 2 μL of loading buffer and heated for 2 min at 45 °C prior to electrophoresis in 1% agarose gels in 40 mM Tris-acetate (pH 8.3) and 2 mM EDTA containing 0.5 μg/mL ethidium bromide. DNA bands were visualized by midrange ultraviolet light and quantified using an Alpha Innotech digital imaging system (Protein Simple). Double-stranded DNA cleavage was monitored by the conversion of negatively supercoiled plasmid molecules to linear plasmid molecules. CC_50_ values (the concentration of drug that induced 50% maximal DNA cleavage complex formation) were calculated on GraphPad Prism Version 10.0.3 using a nonlinear regression analysis with 95% confidence intervals.

For the assay that monitored competition between ciprofloxacin and 8-methyl-2,4-quinazolinedione with GyrA^S91F/D95G^ gyrase, the level of double-stranded DNA cleavage generated by 500 μM ciprofloxacin in the absence of quinazolinedione was used as a baseline and was subtracted from the cleavage level seen in the presence of both compounds. The amount of double-stranded DNA scission observed in the presence of 50 μM 8-methyl-2,4-quinazolinedione alone was set to 100% to directly compare the ability of ciprofloxacin to compete with the quinazolinedione in the active site of the enzyme. Ciprofloxacin (0–500 μM) and 8-methyl-2,4-quinazolinedione (50 μM) were added simultaneously to reaction mixtures. IC_50_ values were calculated on GraphPad Prism Version 10.0.3 using a nonlinear regression analysis with 95% confidence intervals.

## Supporting Information Available

Effects of ciprofloxacin on gyrase-catalyzed DNA supercoiling, effects of ciprofloxacin on gyrase-mediated DNA cleavage, effects of ciprofloxacin on topoisomerase IV-catalyzed DNA decatenation, effects of ciprofloxacin on topoisomerase IV-mediated DNA cleavage (PDF)

## Accession Codes

*N. gonorrhoeae* Gyrase, GyrA: UniProtKB P48371, GyrB: UniProtKB P22118; *N. gonorrhoeae* Topoisomerase IV, ParC: UniProtKB P48374, ParE: UniProtKB A0A8D9YA86.
