# Supplementary material for: Target-Mediated Fluoroquinolone Resistance in Neisseria gonorrhoeae: Actions of Ciprofloxacin against Gyrase and Topoisomerase IV
Source: ACS Infect Dis. 2024 Mar 4;10(4):1351–60. doi: 10.1021/acsinfecdis.4c00041 (PMC11015056; doi:10.1021/acsinfecdis.4c00041)
Supplement: Supplementary file 1 — id4c00041_si_001.pdf [file id4c00041_si_001.pdf]

Supporting Information

Target-Mediated Fluoroquinolone Resistance in *Neisseria gonorrhoeae*: Actions of Ciprofloxacin against Gyrase and Topoisomerase IV

Jessica A. Collins[†], Alexandria A. Oviatt[†,#], Pan F. Chan[§], and Neil Osheroff[†,‡,*]

Departments of [†]Biochemistry and [‡]Medicine (Hematology/Oncology), Vanderbilt University School of Medicine, Nashville, TN 37232, USA; [§]Infectious Diseases Research Unit, GlaxoSmithKline, Collegeville, PA 19426, USA

[#]Present Address: Department of Microbiology and Molecular Genetics, Michigan State University, East Lansing, MI 48824, USA

*To whom correspondence should be addressed. Email: neil.osheroff@vanderbilt.edu. Telephone: +1-615-322-4338

Figure S1

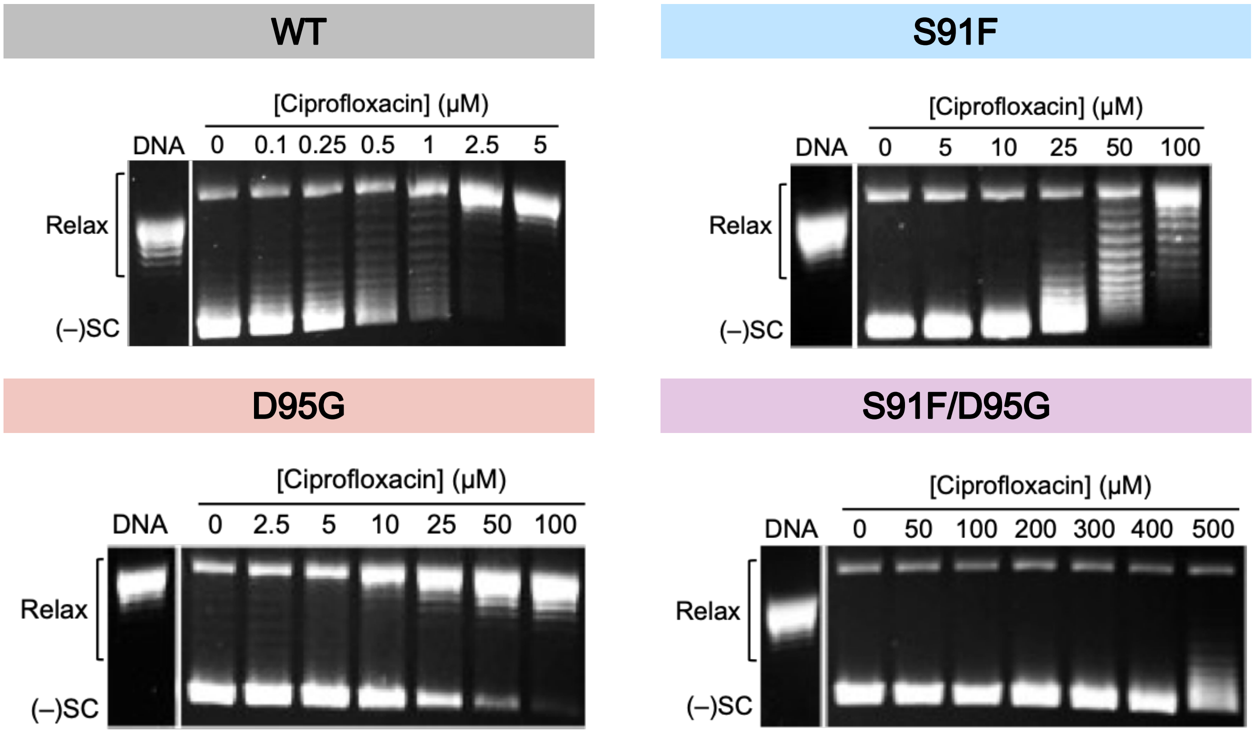

Figure S1. Effects of ciprofloxacin on DNA supercoiling catalyzed by WT and mutant *N. gonorrhoeae* gyrase. The abilities of WT (gray), GyrA^{S91F} (S91F, blue), GyrA^{D95G} (D95G, red) and GyrA$^{S91F/D95G}$ (S91F/D95G, purple) gyrase to supercoil relaxed plasmid (DNA) in the presence of ciprofloxacin are shown. The positions of relaxed (Relax) and negatively supercoiled [(–)SC] plasmid are indicated. Each gel is representative of 3 independent experiments. It should be noted that the relaxed DNA control lane (DNA) was located on the same gel, but several lanes away from the 0 μM ciprofloxacin lane. Intermediary lanes, which included a compound that was not relevant to the present studies, were removed for the sake of clarity.

Figure S2

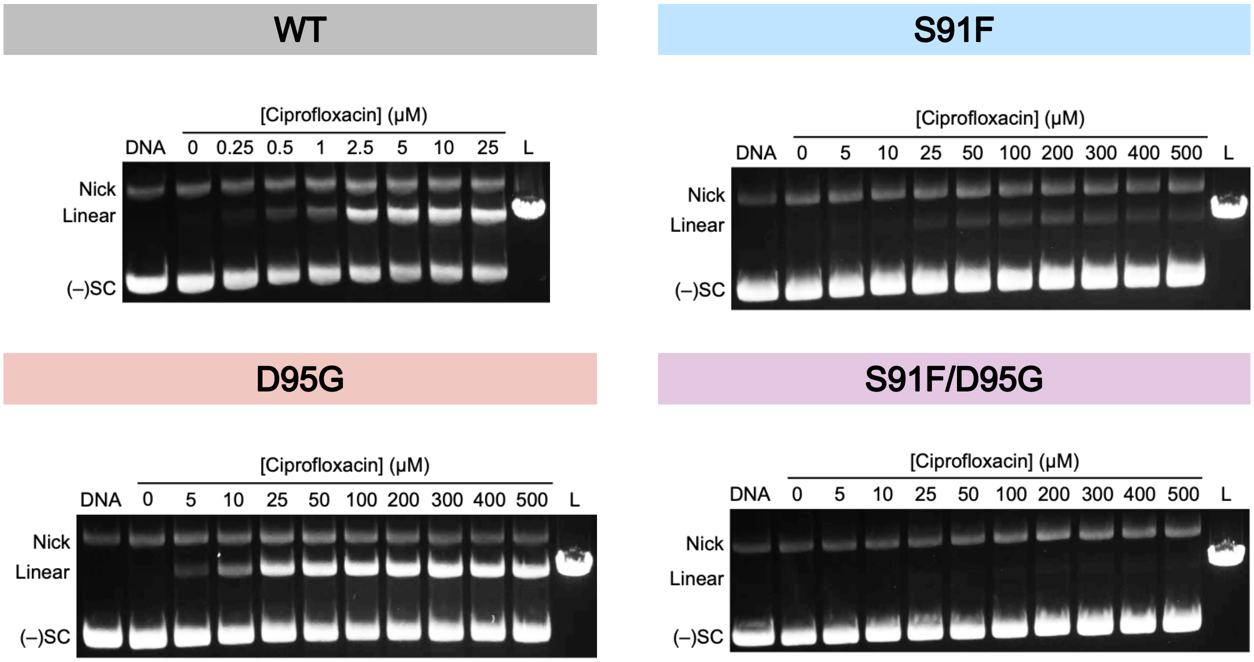

Figure S2. Effects of ciprofloxacin on DNA cleavage mediated by WT and mutant *N. gonorrhoeae* gyrase. The ability of ciprofloxacin to induce double-stranded DNA cleavage mediated by WT (gray), $GyrA^{S91F}$ (S91F, blue), $GyrA^{D95G}$ (D95G, red), and $GyrA^{S91F/D95G}$ (S91F/D95G, purple) gyrase is displayed. Negatively supercoiled (DNA) and linear (L) plasmids are included as controls. The positions of nicked (Nick), linear (Linear), and negatively supercoiled [(–)SC] plasmid are indicated. Each gel is representative of 3 independent experiments.

Figure S3

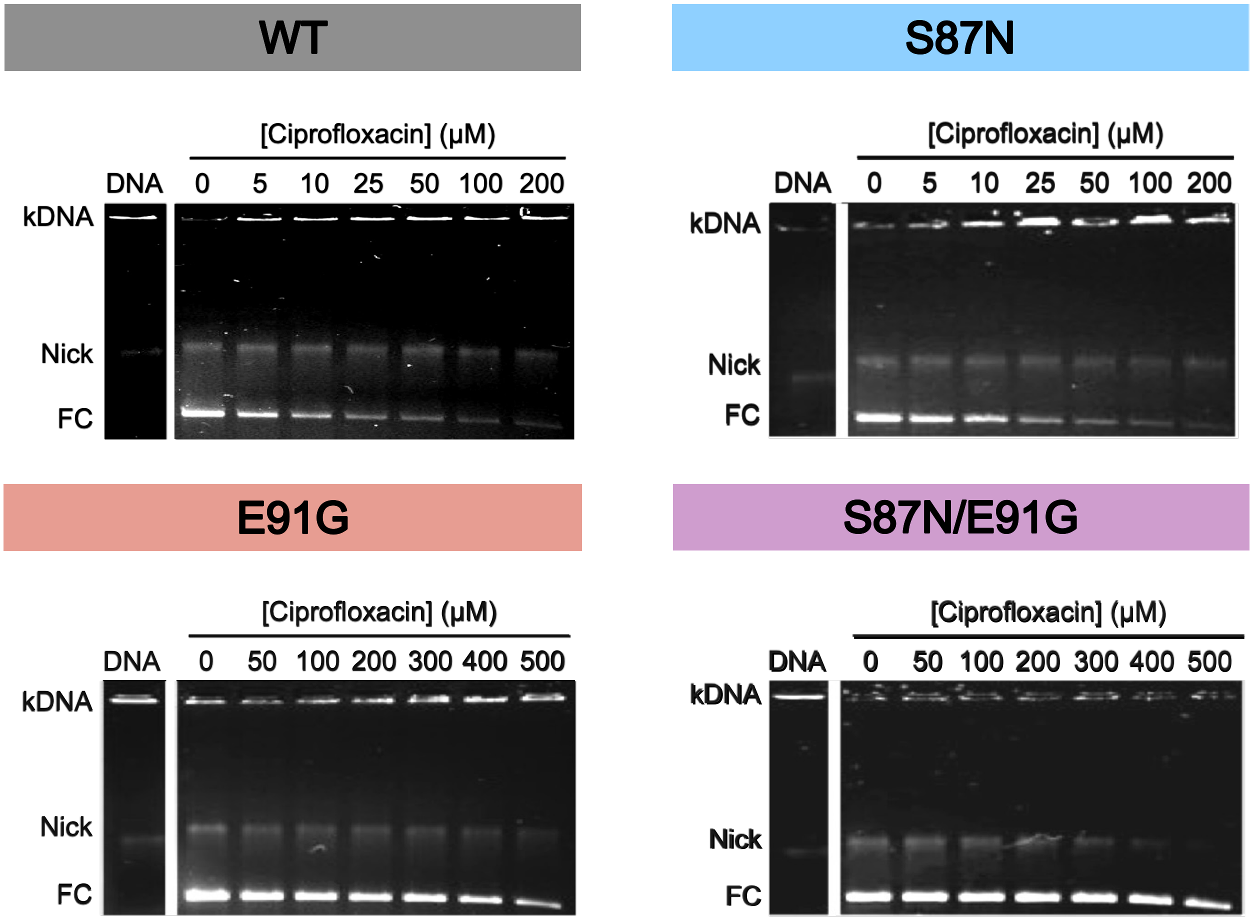

Figure S3: Effects of ciprofloxacin on the DNA decatenation activities of WT and mutant *N. gonorrhoeae* topoisomerase IV. The ability of ciprofloxacin to inhibit decatenation catalyzed by WT (gray), ParC^{S87N} (S87N, blue), ParC^{E91G} (E91G, red), and ParC$^{S87N/E91G}$ (S87N/E91G, purple) topoisomerase IV is shown. The positions of catenated DNA (kDNA), nicked (Nick), and monomeric free circle (FC) plasmids are indicated. Each gel is representative of 3 independent experiments. It should be noted that the catenated kDNA control lane (DNA) was located on the same gel, but several lanes away from the 0 μM ciprofloxacin lane. Intermediary lanes, which included a compound that was not relevant to the present studies, were removed for the sake of clarity.

Figure S4

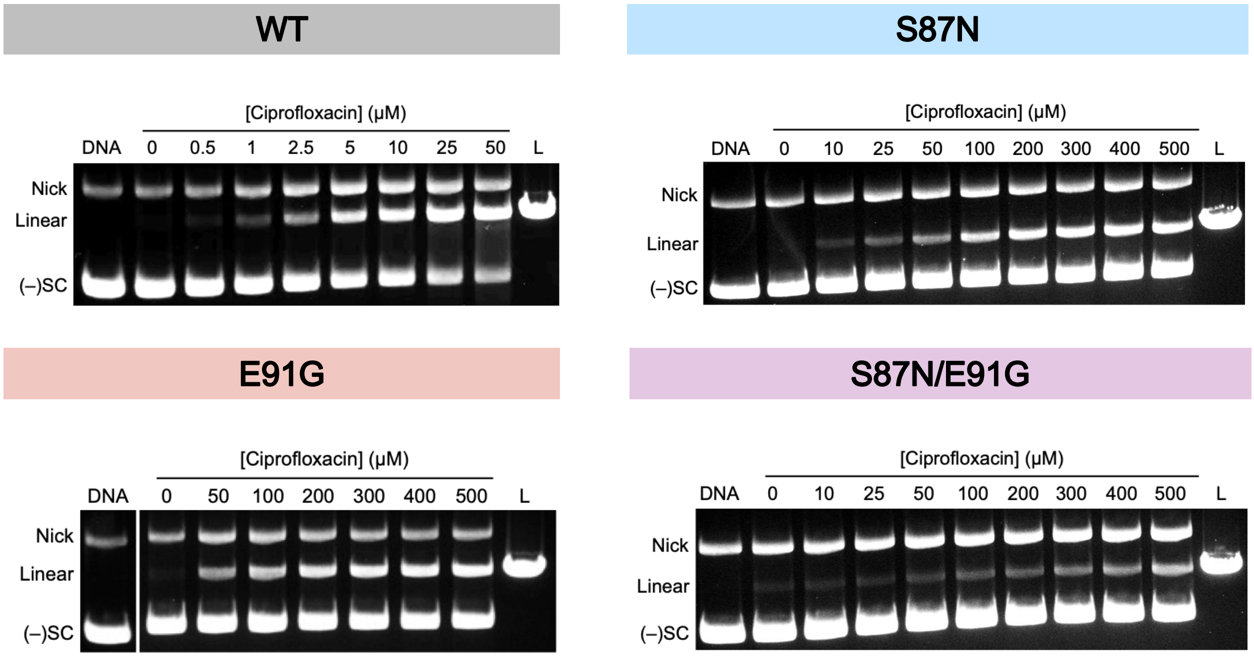

Figure S4: Effects of ciprofloxacin on the DNA cleavage activities of WT and mutant *N. gonorrhoeae* topoisomerase IV. The ability of ciprofloxacin to induce double-stranded (DS) DNA cleavage mediated by WT (gray), ParC^{S87N} (S87N, blue), ParC^{E91G} (E91G, red), and ParC$^{S87N/E91G}$ (S87N/E91G, purple) topoisomerase IV is displayed. Negatively supercoiled (DNA) and linear (L) plasmids are included as controls. The positions of nicked (Nick), linear (Linear), and negatively supercoiled [(–)SC] plasmid are indicated. Each gel is representative of 3 independent experiments. It should be noted that on one of the gels, the negatively supercoiled DNA control lane (DNA) was located on the same gel, but several lanes away from the 0 μM ciprofloxacin lane. Intermediary lanes, which included a compound that was not relevant to the present studies, were removed for the sake of clarity.
